# Comparing the effects of transcranial alternating current and temporal interference (tTIS) electric stimulation through whole-brain mapping of c-Fos immunoreactivity

**DOI:** 10.3389/fnana.2023.1128193

**Published:** 2023-03-13

**Authors:** Venezia G. Carmona-Barrón, Inés S. Fernández del Campo, José M. Delgado-García, Antonio J. De la Fuente, Ignacio Plaza Lopez, Miguel A. Merchán

**Affiliations:** ^1^Institute of Neuroscience of Castilla y Leon (INCYL), University of Salamanca, Salamanca, Spain; ^2^Neuroscience Division, University Pablo de Olavide (UPO), Sevilla, Spain

**Keywords:** rat - brain, GFAP - glial fibrillary acidic protein, IBA1 - ionized calcium binding adaptor molecule 1, blood brain barrier (BBB), independent component analysis (ICA transform), deep brain simulation

## Abstract

The analysis of the topography of brain neuromodulation following transcranial alternating current (AC) stimulation is relevant for defining strategies directed to specific nuclei stimulation in patients. Among the different procedures of AC stimulation, temporal interference (tTIS) is a novel method for non-invasive neuromodulation of specific deep brain targets. However, little information is currently available about its tissue effects and its activation topography in *in vivo* animal models. After a single session (30 min, 0.12 mA) of transcranial alternate current (2,000 Hz; ES/AC group) or tTIS (2,000/2,010 Hz; Es/tTIS group) stimulation, rat brains were explored by whole-brain mapping analysis of c-Fos immunostained serial sections. For this analysis, we used two mapping methods, namely density-to-color processed channels (independent component analysis (ICA) and graphical representation (MATLAB) of morphometrical and densitometrical values obtained by density threshold segmentation. In addition, to assess tissue effects, alternate serial sections were stained for glial fibrillary acidic protein (GFAP), ionized calcium-binding adapter molecule 1 (Iba1), and Nissl. AC stimulation induced a mild superficial increase in c-Fos immunoreactivity. However, tTIS stimulation globally decreased the number of c-Fos-positive neurons and increased blood brain barrier cell immunoreactivity. tTIS also had a stronger effect around the electrode placement area and preserved neuronal activation better in restricted areas of the deep brain (directional stimulation). The enhanced activation of intramural blood vessels’ cells and perivascular astrocytes suggests that low-frequency interference (10 Hz) may also have a trophic effect.

## Highlights

– Transcranial temporal interference stimulation (tTIS) decreases neural activation
– tTIS induces deep, directional activity neuromodulation
– Vessel cells increased immunoreactivity suggest hemodynamic changes after tTIS.

## 1. Introduction

Direct current (DC) stimulation modulates spontaneous neuronal activity in a polarity-dependent way, with site-specific effects. In DC stimulation, penetration, and direction depend on the conductance of neural tissue liquids and barriers. Recordings performed in rats after anodal DC stimulation with trains of pulses delivered to the skin, skull, and dura have demonstrated that voltage declines quickly in the brain towards a low global value of conductivity of 0.57 S/m (Asan et al., 2019). Due to physical interactions between electric currents and neural tissues, DC stimulation induces a limited activation of the cortex, which is less than 1 mm deep, as previously shown in rat animal models (Colmenárez-Raga et al., 2019; Díaz et al., 2021). Alternating current (AC) stimulation, in contrast, does not induce polarizing effects but activates neuronal networks *via* rhythmic stimulation by synchronizing their endogenous neurophysiological activities (Fröhlich and McCormick, 2010; Zaehle et al., 2010; Reato et al., 2013; Abd Hamid et al., 2015; Vossen et al., 2015). Intracranial electrophysiological recordings in rats, stimulated with sinusoidal currents using surface electrodes at intensities as low as 1 V/m, show synchronization of hippocampal neurons, suggesting that noninvasive AC stimulation may be a suitable method for deep and safe brain stimulation (Ozen et al., 2010). However, focal activation of defined areas of the brain requires other strategies, some of which have been tested in the last decade. Among these recently developed AC stimulation procedures, one of the most promising methods for deep non-invasive stimulation is transcranial temporal interference stimulation (tTIS). tTIS has stood out in recent contributions for its effectiveness demonstrated in biophysics computational models (Huang and Parra, 2019; Rampersad et al., 2019; Mirzakhalili et al., 2020) and for its effects on the brain assessed by *in vivo* anatomical and functional analysis (Grossman et al., 2017). Notwithstanding its recent advances, the underlying principle of tTIS is not new. In fact, interferential therapy was first proposed in 1950 by Hans Nemec towards avoiding the discomfort induced by low-frequency currents in the skin during electrotherapy. In this method, simultaneous activation of two AC circuits, with offset frequencies, induces an interactive field, resulting in low-frequency current amplification in the tissue (for a historical review please refer to Ganne, 1976; Goats, 1990). But despite having been used and improved for electrotherapy purposes over the years (Ozcan et al., 2004; Fuentes et al., 2010; Beatti et al., 2011, 2012; Santos et al., 2013; Mendonça Araújo et al., 2017), stimulation *via* temporally interfering electric fields has only been recently explored as a method for brain neuromodulation. Currently, the available evidence on interferential currents applied to the brain mostly derives from studies involving human and murine computational models (Huang and Parra, 2019; Rampersad et al., 2019; Mirzakhalili et al., 2020).

In humans, electrode configuration changes have been tested to optimize tTIS. For example, human head modeling for multi-electrode optimization has demonstrated the potential of AC for generating guiding currents through the cerebrospinal fluid (CSF) and brain blood vessels (Soldozy et al., 2020). However, functional magnetic resonance imaging (fMRI) has indicated correlation coefficients between patients ranging from 0.57 to 0.94, underscoring the high variability of electric field distributions across individuals (von Conta et al., 2021). Moreover, this variability in tTIS effects between patients may depend not only on the position of the electrodes and on the shape and size of the head but also on differences in the anisotropy and in the conductivity of the brain barriers that the electric current must cross. The need for a better understanding of the blood brain barrier reactions when electric currents cross blood vessel walls has been highlighted by new, non-invasive deep brain stimulation strategies in which currents are delivered by endovascular multi-electrode stent implantation (Soldozy et al., 2020). Therefore, further research must be conducted to assess the effects of temporal interference stimulation on tissues and their activation topography in *in vivo* animal models.

Considering the above, our study aims to comprehensively map neural and glial activation induced by tTIS to elucidate how electric fields travel across and activate different areas of the brain. For this purpose, we used three immunocytochemical markers, namely c-Fos, glial fibrillary acidic protein (GFAP), and ionized calcium-binding adapter molecule 1 (Iba1; Dragunow et al., 1987; Sheng and Greenberg, 1990; Herrera and Robertson, 1996). c-Fos has been successfully used to determine the neuronal effects of tTIS in the central nervous system (da Silva et al., 2014; Grossman et al., 2017). In our laboratory, we have used c-Fos as an activity marker as well as MATLAB mapping in previous experimental approaches (Clarkson et al., 2010; Lamas et al., 2017; Pernia et al., 2017, 2020). And while our non-invasive approach should not induce severe tissue damage, we also assessed microglial reactivity by measuring Iba1 immunocytochemistry and neuronal preservation through Nissl staining.

## 2. Materials and methods

This study was conducted in strict accordance with Spanish regulations (Royal Decree 53/2013—Law 32/2007) and European Union guidelines (Directive 2010/63/EU) on the care and use of animals in biomedical research (Permit number: USAL- 685-2021). All surgeries were performed under monitored anesthesia (respiratory rate, body temperature, and oxygen saturation), and all efforts were made to minimize animal suffering. In total, 15 young male Wistar rats weighing from 200–250 g were randomly separated into three experimental groups, namely Sham Controls (SC; *n* = 5), Electrical Stimulation/transcranial Temporal Interference Stimulation (ES/tTIS; *n* = 5), and Electrical Stimulation/Alternating Current (non-interference) stimulation (ES/AC; *n* = 5) groups.

### 2.1. Surgery and electrical stimulation

Our tTIS protocol was based on the experimental design of Grossman et al. (2017), applied to c-Fos immunocytochemistry analysis. Rats were placed in a stereotaxic frame, and under gas anesthesia with 2.5% isoflurane, their skull surface was surgically exposed. The scalp and the ventral region of the neck were shaved and sterilized with betadine and 70% ethanol, and the eyes were protected with ophthalmic drops (Methocel^®^ 2%, OmniVision Technologies Inc., Santa Clara, CA, USA).

After exposing the skull, two points were labeled at the left side of the skull at 4.56 mm caudal to Bregma, and 1 mm and 3.85 mm lateral (Paxinos and Watson, 1998). Two polyamide tubes (1.00 and 0.9 mm in outer and inner diameters, respectively; Poly Medicure Ltd., New Delhi, India) were centered on the points and glued with dental cement. To ensure that the cement had not penetrated the tubes, they were carefully checked. The tubes were then filled with saline, and two silver wires 0.20 mm in diameter (AM Systems^®^ #786500, Sequim, WA, USA) were placed inside them. Two self-adhesive electrodes (11 mm in diameter of the conductive area; EL504, BioPac Systems Inc., Goleta, CA, USA) with conductive electrode gel (SignaGel, Parker Laboratories Inc, Fairfield, NJ, USA) were glued in the ventral part of the head, at 4 mm from the interaural line.

In the following experimental step, and under gas anesthesia, the rats were stimulated with 0.12 mA pulses, presented with a 10-s-on/10-s-off cycle in 0.5-s ramps. Cranial electrodes were paired in parallel, and the entire session lasted for 30 min. The ES/AC group was stimulated at 2,000 Hz in both circuits. Conversely, in the ES/tTIS group, the lateral circuit was activated at 2,000 Hz and the medial circuit at 2,010 Hz. Stimulations were performed using a CS-420 programmable wave generator equipped with two ISU-300 isolation units (Cibertec, Madrid, Spain). The current intensity of the signals F_1_I_1_ and F_2_I_2_ was controlled through the isolation units, which were connected to different channels of the stimulator. To ensure that both F_1_I_1_ and F_2_I_2_ currents were presented simultaneously to the animal, we connected a CS-420 digital stimulator to an external trigger (CS-220, Cibertec, Madrid, Spain). Before each stimulation, we verified that the equipment worked properly and generated the expected output signals according to the configured parameters using a digital oscilloscope (Tektronix TDS 2014B from Tektronic, Beaverton, Oregon, USA). After the stimulation session, we returned the animals to their cages for the next 40 min before euthanasia. The sham controls were processed similarly, albeit without electric stimulation.

### 2.2. Histology

Forty minutes after electrical stimulation, the animals were deeply anesthetized with an intraperitoneal injection of 6% sodium pentobarbital [60 mg/kg body weight (BW)] and perfused transcardially with 4% p-formaldehyde in a 0.1-M phosphate buffer saline PBS. After fixation, the brains were dissected out, sectioned, and postfixed by immersion in the same fixative solution for 2 h before being cryoprotected by immersion in 30% sucrose in 0.1 M PBS, pH 7.4 at 4°C for 48 h. The brains were then serially sectioned in the coronal plane at 40 μm using a sliding freezing microtome (Overall number of sections per animal = 336 +/– 10). Alternate serial sections were stained with Nissl and incubated in primary antibody for anti c-Fos (1:1,000- Synaptic Systems #226003, Göttingen, Germany); anti-GFAP (1:500 - Sigma #G6171, Darmstadt, Germany) and anti-Iba1 (1:1,000 Fujifilm Wako Pure Chemical Corporation #019-19741, Osaka, Japan) in Tris-buffered saline with 1% Triton X-100 (TBS-Tx). After 48 h, the sections were washed and incubated in an anti-rabbit biotinylated secondary antibody (biotinylated anti-rabbit IgG H1L, BA-1000; Vector, Burlingame, CA, USA) and in an anti-mouse biotinylated secondary antibody (biotinylated anti-rabbit IgG H1L, BA-2000; Vector, Burlingame, CA, USA) at a 1:200 dilution in TBS-Tx for 120 min at room temperature. The sections were then washed with TBS-Tx and incubated for 180 min in avidin/biotin–peroxidase (ABC complex, Vectastain Standard ABC kit PK-4000; Vector, Burlingame, CA, USA). Lastly, the sections were placed in 3, 3-diaminobenzidine tetrahydrochloride (DAB; D-9015; Sigma-Aldrich, St. Louis, MO, USA) with 0.006% H_2_O_2_ and 0.3% nickel ammonium sulfate for 10 min and washed with a Tris-HCl solution before mounting and cover slipping. As a control for immunostaining, a section of each experimental case was incubated in TBS-Tx without the primary antibody, showing no detectable reactivity.

### 2.3. Image analysis

High-resolution microphotographs (digital resolution of 132 pixel/100 μm^2^) were taken at 10× magnification under a Leica DMRX microscope with an MBF camera (MBF Bioscience CX9000; Williston, VT, USA) to prepare whole-section digital mosaics using the Neurolucida software (NL-Vs 8.0, MicroBrightField^®^, Inc., Williston, VT, USA). To set homogeneous microscopic illumination conditions and to calibrate optical density (OD) measurements, photographs were taken using a standardized grayscale range and a stepped density filter (11 levels; ^®^EO Edmund industrial optics-ref 32599, Karlsruhe, Germany).

For morphometrical analysis, immunoreactive neurons were segmented by density thresholding using ImageJ software and the Maximum Entropy plug-in. Thus, the values of coordinates, area, and optical density of the segmented cells were collected to perform statistical analysis and to build the c-Fos activation maps (MATLAB). Normalized OD values of immunoreactive cells were calculated by subtracting the mean OD of the whole section from the OD values of segmented particles, divided by the gray standard deviation of the whole slice. The number of segmented cells was normalized to N/10,000 μm^2^ of surface area.

### 2.4. Brain mapping

#### 2.4.1. Independent component analysis (ICA) maps

To improve the direct visualization of interstitial reaction products, a pseudocolor filter of the independent component analysis (ICA) color space method was applied (ICA3 ImageJ Plugin). This method separates an image into three independent and uncorrelated color components or sources, namely C1, C2, and C3, which are more effective than R, G, and B component images for pattern recognition, thereby increasing the visual discrimination of density values. A density calibration bar for ICA3, available in ImageJ, was processed in parallel with the images for a consistent visual appreciation of changes between sections or cases. The original captured images were used for this analysis without changing the brightness or contrast.

#### 2.4.2. MATLAB maps

Extensive full-sections maps encoding the location, area, and density of segmented particles were made using MATLAB software (© MATLAB R-2017, Scatterplot function). Optical density was converted into color with the MATLAB color scale Jet from the Scatterplot function.

### 2.5. Statistical analysis

Statistical analysis was performed using IBM^®^ SPSS^®^ software, version 26.0.0.2 for Mac (IBM Corp. and SPSS Inc., Chicago, IL, United States, RRID: SCR_002865). The Shapiro-Wilk test was used to analyze the normal distribution of the values. After checking the normal distribution of the data, the one-way ANOVA test was then used to compare the number of normalized immunoreactive particles between groups. Significant differences between values were analyzed using the *post-hoc* Bonferroni test. Differences were considered statistically significant at *p* < 0.05.

## 3. Results

### 3.1. c-Fos immunocytochemistry

#### 3.1.1. General map features

**Figure 1:**
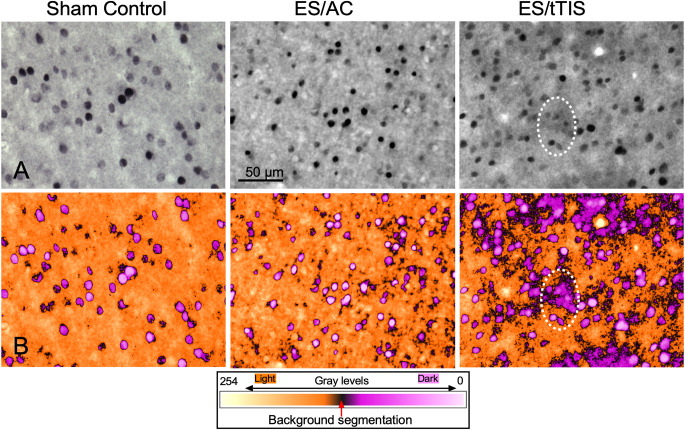
c-Fos immunoreactivity analysis using the independent component analysis (ICA) color space method for pattern recognition. **(A)** c-Fos immunostaining in the three experimental groups. A mild interstitial dense reaction product around the cell nuclei (dotted line) can be observed. **(B)** Corresponding images resulting after optical density-to-color processing applying the ICA3 plugin from ImageJ program. The encircled area (dotted line) shows that nuclei in white can be easily distinguished from the cytoplasm in the magenta. All images were taken under the same conditions of microscopic illumination and analyzed without any digital manipulation of brightness, contrast, or color. The inset shows the optical density-to-color calibration bar.

The ICA maps showed dense interstitial immunostaining around the nuclei in the ES/tTIS group (Figure 1B, dotted line). No interstitial immunoreactive product was detected in white matter or in areas without positive cells (not shown). Thus, pseudocolor enhancement with the ICA3 plugin highlighted these subtle differences difficult to perceive by conventional microscopy observation (Figure 1). In addition, after color transformation, immunoreactive areas with density values just below those of nuclei were identified as cell cytoplasm in magenta, which were more evident and larger in ES/tTIS (Figure 1B). These results indicate that our ICA analysis differentiated c-Fos-immunostained cytoplasm from the nuclei.

**Figure 2:**
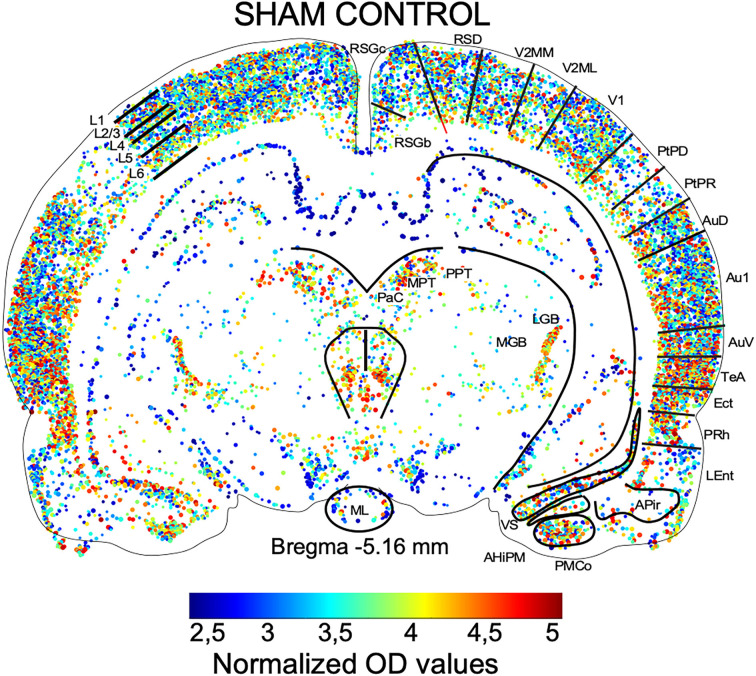
Brain map after MATLAB reconstruction from segmented c-Fos immunostained sections. The lines and cytoarchitectonic subdivisions are modified from Paxinos and Watson’s rat brain atlas (Paxinos and Watson, 1998). Superimposing the atlas and the MATLAB map at the Bregma -5.16 mm section with cytoarchitectonic subdivisions of the cortex and brain stem nuclei highlights topographical differences in the distribution of dots and colors (normalized OD values). L- cortical layers. Abbreviations of cytoarchitectonic subdivisions follow the nomenclature of the Paxinos and Watson’s rat brain atlas (Paxinos and Watson, 1998).

The graphical representation of sizes (encoded as dot areas) and OD values (encoded as colors) of c-Fos immunoreactive cells, assessed by density threshold segmentation, in MATLAB, match the architectonic subdivisions of Paxinos and Watson’s rat brain atlas (Paxinos and Watson, 1998; Figure 2). In our segmental maps, the cortical layers (parallel lines in Figure 2) and subdivisions (orthogonal lines in Figure 2) follow the established neural organization of the rat brain. Therefore, our MATLAB mapping procedure provides a suitable reference for visual analysis of cytoarchitectonic changes after experimental interventions.

For an adequate map interpretation, a suprathreshold electric current stimulation effect should be considered when observing loss or blurring of cytoarchitectonic subdivisions after mapping c-Fos immunostained sections. In addition, skull electrodes were placed unilaterally, on the left side. For this reason, interhemispheric asymmetric changes in dot color, size or clustering were also considered a stimulation effect.

#### 3.1.2. ICA map analysis

**Figure 3:**
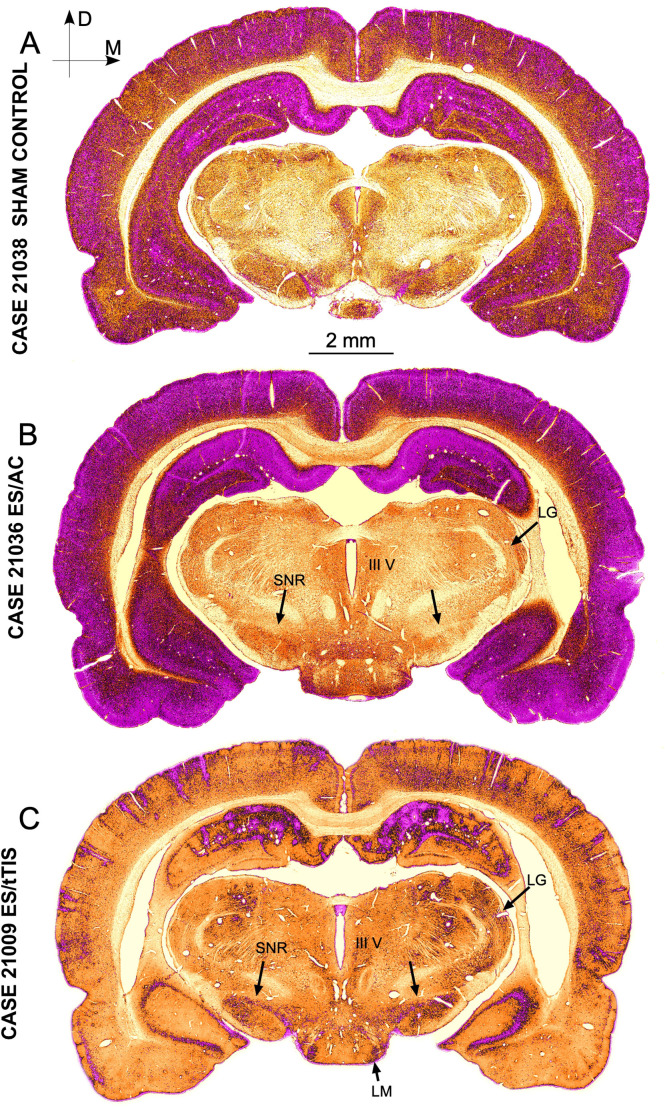
c-Fos immunostained sections after ICA pseudocolor conversion (sections at the electrodes placement coordinates). **(A)** Sham Control. **(B)** ES/AC section showing a homogeneous increase in immunoreactivity in cortical and hippocampal areas. Note the scarce immunostaining in deep brain areas. **(C)** ES/tTIS section showing a global loss of cell immunoreactivity. Note the intense immunoreactive in the cortex and dentate gyrus around vessels and the asymmetric distribution of staining in the ventral hippocampus. Periventricular immunoreactivity and diencephalic nuclei staining are clearly more intense in ES/tTIS than in ES/AC. III V—Third ventricle, LG—Lateral geniculate, LM—Lateral mammillary nucleus, SNR—Substantia nigra pars reticulata.

**Figure 4:**
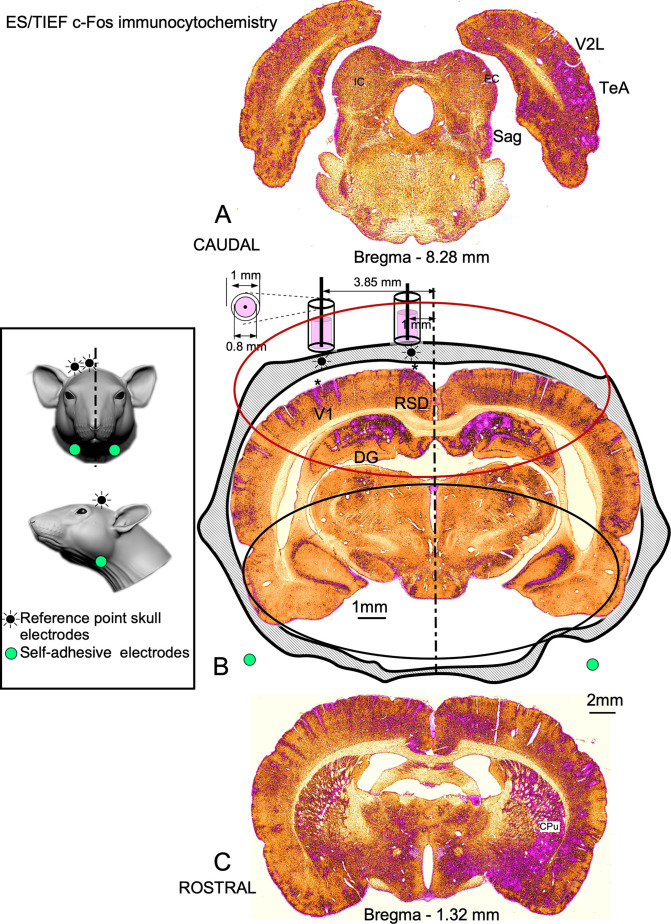
Representative c-Fos immunostaining of ES/tTIS serial coronal sections after ICA pseudocolor conversion. **(A)** Top caudal, **(B)** electrode coordinates and **(C)** top rostral. Perivascular magenta reaction defining asymmetric (denser in the right hemisphere) areas of activation throughout the rostro-caudal axis. **(A)** At Bregma -8.28 mm, denser cell and perivascular immunoreactivity is observed in the right temporal associative (TeA), secondary visual cortex (V2L), external cortex of the inferior colliculus (EC), and nucleus sagulum (Sag). IC, Inferior colliculus. **(B)** In the electrodes, coordinates at Bregma - 4.56 mm, at the level of the retrosplenial cortex (RSD) and visual primary cortex (V1), higher density areas (asterisks) are observed in the left hemisphere, matching the estimated position of the electrodes (cylinders on the top of the skull silhouette). The red oval shape in the dorsal area, under the position of the electrode, indicates preserved staining symmetry. The encircled black ventral area shows an asymmetric staining in the hippocampal region. Inset. Diagram indicating the position of the electrodes in the head of the rat. **(C)** The rostral section at Bregma -1.32 mm showing asymmetric staining laterally in the ventral striatum, in the caudate putamen (CPu), and medially in the anterior hypothalamic area. For a full extensive serial sections visualization please see video in Supplementary Materials.

In the SC group, differences in density allowed us to differentiate cortical and hippocampal layers and subdivisions, as well as weak staining in deep brain areas (Figure 3A). However, in the ES/AC group, immunoreactivity homogeneously increased on the surface, with no differences in immunostaining in the deep brain (Figure 3B). In ES/tTIS maps, fewer immunoreactive neurons and overstained blood vessels were detected at all levels of sectioning (Figure 4, shown in magenta). These stimulation effects were bilateral and maximal at the placement coordinates of the skull electrodes, as observed in a wide dorsal area, more than 3 mm apart from the electrodes (Figure 4B, red oval line). Conversely, far from the electrodes, bilateral differences in immunostaining (asymmetry) were identified both ventrally (Figure 4B, black oval line) and along the whole caudo-rostral axis (Figures 4A–C). Staining in the deep brain (thalamic and hypothalamic areas) was evident in ES/tTIS, but not in ES/AC (please compare the substantia nigra pars reticulata (SNR), the lateral mammillary nucleus (LM) and the lateral geniculate body (LG) in Figures 3B,C.

#### 3.1.3. MATLAB map analysis

**Figure 5:**
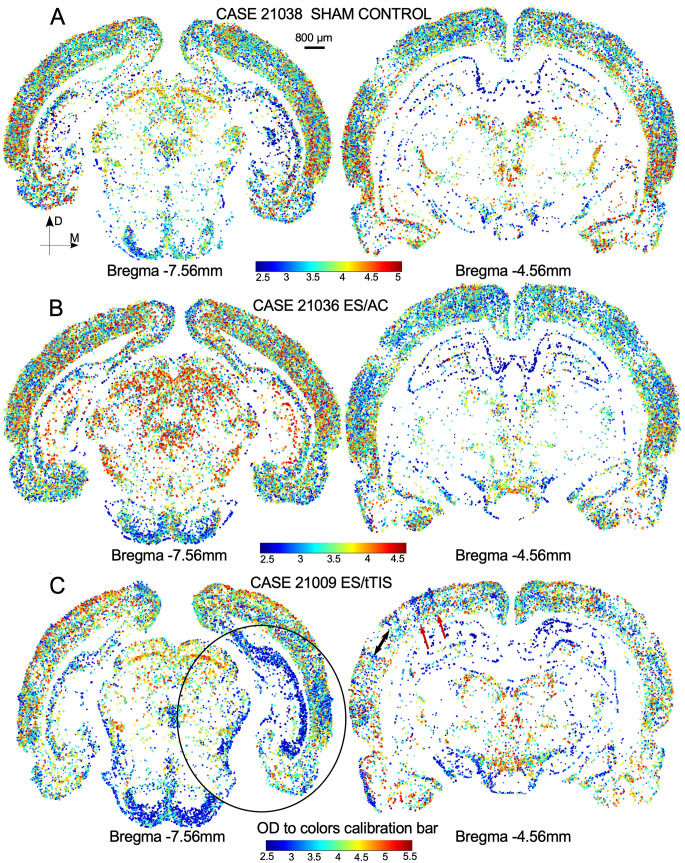
MATLAB maps of two representative sectioning levels (caudal left and rostral right column) from **(A)** Sham Controls, **(B)** ES/AC, and **(C)** ES/tTIS at equivalent coordinates. **(B)** ES/AC maps show the same cytoarchitectonic organization as the maps of sham controls (compare with **A**). **(C)** ES/tTIS maps show a decreased number of cells with areas devoid of staining (black double arrow), particularly at Bregma -4.56 mm (right column). At these coordinates, cortical cytoarchitectonic borders are blurred and orthogonal rows of cells (red arrows) can be seen. At the caudal level (left section Bregma -7.56 mm), the cell population shows an asymmetric distribution, especially in the right hippocampus and around the ventral temporal associative (TeA) cortex (circle). Insets in the bottom of the maps show the optical density-to-color calibration bars.

The analysis of the digital maps showed a well-preserved and symmetric cortical cytoarchitecture, with a random increase in OD along serial sections (increase in red dots) when comparing the ES/AC and sham control group (Figure 5, compare A and B). In ES/tTIS, the number of dots consistently decreased in the cortex of sections around the coordinates of the surface electrode, as shown by empty areas without staining (bald patches; Figure 5C, right, black double arrow). These empty areas were delimited by vertical rows of positive cells, presumably perforating arteries (Figure 5C, right, red arrows). In the ES/tTIS group, the decrease in immunoreactive cells close to the area of electrode placement does not allow to clearly define cortical subdivisions or laminations as in similar coordinates from the other experimental conditions (compare Figures 5A,C). In the most caudal sections, the cortex was populated with dots, showing an asymmetric distribution of staining in the deep areas of the brain (Figure 5C, left, circle).

#### 3.1.4. Quantitative analysis

**Figure 6:**
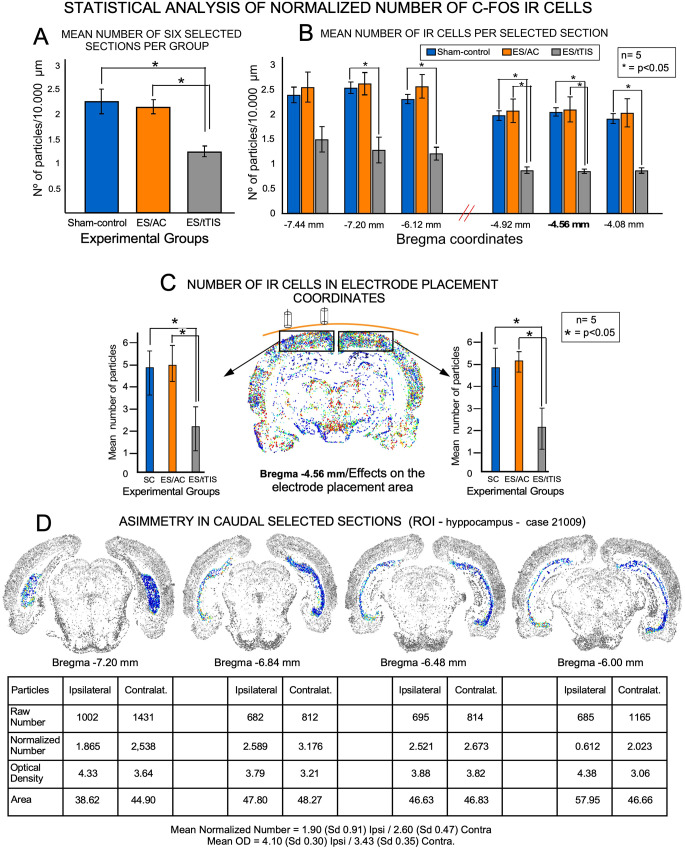
Quantitative analysis of c-Fos immunostained sections. **(A)** Statistical analysis of normalized number of cells from six selected coronal sections per experimental group (*n* = 5). **(B)** Statistical comparison of normalized number of neurons from six sections at equivalent coordinates in the three experimental groups. In bold in the x-axis, (Bregma -4.56 mm) points to the skull electrodes’ placement coordinates. Note that there are no significant differences between experimental groups in caudal sections (Bregma -7.44 mm), far from the electrode localization. **(C)** Statistical analysis of the normalized number of neurons in the dorsal area of the cortex (rectangles in the map indicate the selected ROIs) after comparing Bregma -4.56 mm sections between groups (five ROIs from left and five ROIs from the right hemisphere for each experimental group). Significant differences are observed after comparing SC (blue columns) or ES/AC (orange columns) with ES/tTIS groups (gray columns). **(D)** Side-by-side comparison of parameters from segmented cells (number of cells, OD, and area) in an ES/tTIS selected case. ROI in blue in the silhouettes (Subiculum). Asymmetric immunostaining is shown by numerical differences in neurons (table at the bottom of the figure).

After density threshold segmentation, graphically represented morphometrical data were quantitatively analyzed (Figure 6). When comparing the normalized number of immunoreactive cells from six selected coronal serial sections (at equivalent coordinates) together, significant differences in the means were found between the SC and tTIS (*p* = 0.021) and between ES/AC and tTIS experimental groups (*p* = 0.019; Figure 6A). When comparing at different caudo-rostral levels the mean values of normalized cells from six selected sections separately a significant decrease of immunoreactive cells in the ES/tTIS group respect to SC (*p* = 0.025) and ES/AC (*p* = 0.031) were detected in the electrode placement (Bregma -4.56 mm) and adjacent sections coordinates (-4.92 and -4.08 mm; Figure 6B). In caudalmost sections (-7.44 mm) no significant differences were observed between groups (Figure 6B).

When comparing defined regions of interest of the cortex (ROIs; rectangles in Figure 6C), no significant differences in the number of normalized c-Fos neurons were found between ES/AC and SC in electrode placement coordinates (Figure 6C). However, a significant, symmetric, and bilateral decrease in the normalized number of cells was assessed when comparing ES/tTIS with SC (ipsilateral *p* = 0.037; contralateral *p* = 0.042) or ES/AC (ipsilateral *p* = 0.025; contralateral *p* = 0.017; Figure 6C).

ES/tTIS sections had noticeable interhemispheric asymmetries in hippocampal and latero-ventral areas of the cortex, among other areas (Figure 4). As an example in Figure 6D, for a given ES/tTIS case, the number of normalized neurons was much higher (up to a 27%) in the contralateral than in the ipsilateral hippocampus at four caudal levels of sectioning.

#### 3.1.5. Blood vessels analysis following c-Fos and GFAP immunostaining

**Figure 7:**
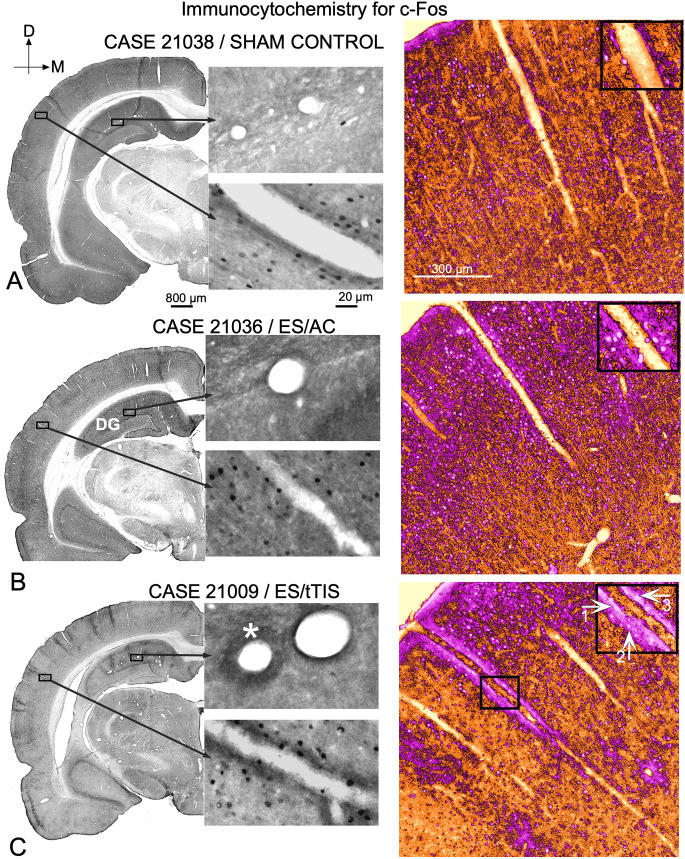
c-Fos immunostaining of blood vessels. **(A)** SC group.** (B)** ES/AC shows increased immunoreactivity in superficial cortical layers of the cortex **(C)** ES/tTIS stained sections show increased immunoreactivity in blood vessels cells. Left column c-Fos immunostaining, non-modified pictures; right column—higher magnification details after ICA color conversion. Insets show details of blood vessels’ immunoreactivity in the three experimental conditions. In the inset in **(C)** white arrows point: **1-** Endothelium; **2-** Smooth muscle cell and **3-** Pericytes.

Our c-Fos-immunostained sections showed well-defined, round, or oval cell nuclei, belonging to either neurons or blood vessel cells (Figure 7). In ES/AC, a homogeneous increase in immunoreactivity was detected by ICA analysis, with slight changes in density and thickness in blood vessel walls (Figure 7B, right, inset). But ES/tTIS, c-Fos-immunostained sections (Figure 7C, left), and ICA maps showed increased immunoreactivity in the blood vessels’ wall (Figure 7C, right). Reactive cells were identified as smooth muscle cells, pericytes, and endothelial cells (Figure 7C, right-inset arrows).

**Figure 8:**
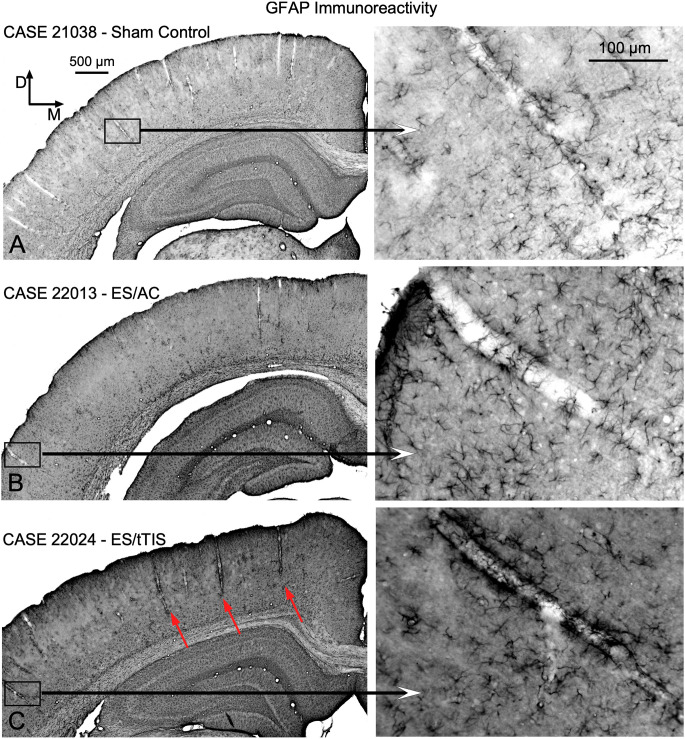
GFAP immunostaining in sections from the area close to the electrode’s coordinates (Bregma -4.56 mm).** (A)** In SC experimental group light stained immunoreactive astrocytes can be observed around blood vessels. **(B)** Slight increase in immunoreactive astrocytes can be seen in ES/AC group. **(C)** Dense reinforcement of immunoreactive astrocytes can be seen around vessels. Red arrows in the panoramic view on the left, point to the distribution of dense stained vessels along the cortex.

GFAP immunostaining shows light labeling of astrocytes around blood vessels in the SC group (Figure 8A), while in the ES/AC group, a slight increase of immunoreactive astrocytes can be observed (Figure 8B). ES/tTIS GFAP-immunostained sections (Figure 8C) showed a marked increase in density of astrocytes around vessels, particularly in areas close to the skull electrode coordinates (Figure 8C, red arrows).

### 3.2. Iba1 and Nissl staining

**Figure 9:**
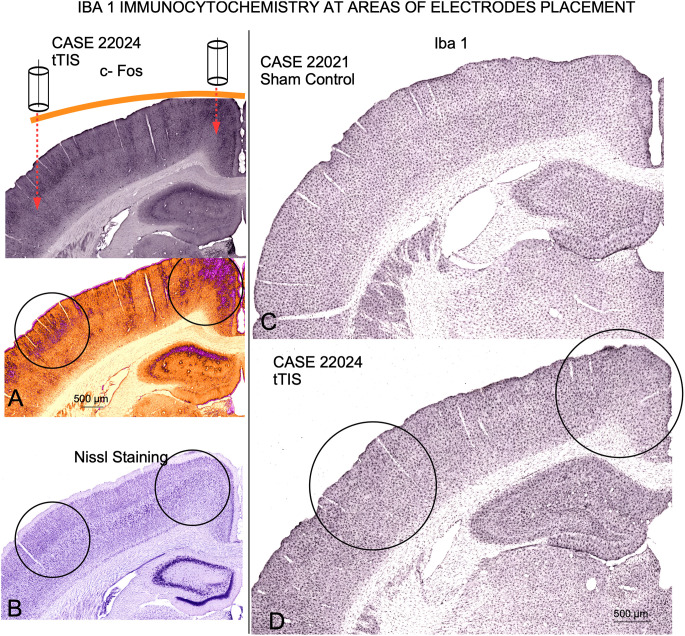
Nissl staining and Iba1 immunoreactivity at the electrode placement coordinates. **(A)** c-Fos immunoreactivity and ICA pseudo color transformation in tTIS experimental case, for localization of reactive areas induced by current stimulation (circles). **(B)** Nissl staining of an adjacent serial section to sections shown in **(A)**. No chromatolysis, loss of neurons or architectural changes were detected. **(C)** Sham control Iba 1 immunostained section. **(D)** tTIS Iba 1 immunostained section at the coordinates of electrode placement. Note that glial architecture is well preserved along the cortex.

No loss of neurons or chromatolytic changes were detected in Nissl-stained sections from ES/tTIS in the area of electrode placement (Figures 9A,B, circles). Also, no substantial changes were observed in any sections analyzed and in any experimental groups after Iba1 immunostaining (Figures 9C,D).

## 4. Discussion

In this study, brain mapping of c-Fos-, GFAP, and Iba1-immunostained serial sections showed the tissue effects of a single session of tTIS (ES/tTIS group) on the rat brain. tTIS was induced using two circuits with paired electrodes at frequencies of 2,000 or 2,010 Hz and at 0.12 mA. In the ES/AC group, conversely, the animals were stimulated at a single frequency of 2,000 Hz in both circuits as controls to assess specific effects of stimulation *via* temporally interfering electric fields. When compared with SC, ES/AC-stimulated rats showed a homogeneous global increase in c-Fos immunoreactivity (magenta shift) in pseudocolor maps prepared using the ICA color space method and a similar number of immunoreactive cells. Thus, AC has mild, shallow, and homogeneous effects on the rat brain.

In the ES/tTIS group, in contrast, a global decrease in immunoreactivity was observed in the brain maps, with a significantly lower number of immunoreactive c-Fos cells, indicating a general decrease in neuronal activation (Figure 6A). In both MATLAB and ICA topographical maps, the maximum loss of immunoreactive cells was located in areas close to the electrode coordinates. Along the brain sections changes in immunoreactivity became gradually milder with the increase in the distance from the position of the electrodes, defining a decreased activation from the electrode placement zones (Figure 6B). The asymmetric configuration of the electrodes on the left side of the skull also resulted in a lower decrease of c-Fos immunoreactivity in deep contralateral brain areas. These findings suggest that acute tTIS induces electric fields with deep, gradual, and directional (vectorial) effects on the brain, decreasing neuronal activation. Moreover, c-Fos-immunoreactive intramural blood vessels and astroglia denote potential trophic effects of temporally interfering electric fields, which should be carefully analyzed in future studies.

As reported in our previous studies, Iba1 immunostaining of microglial cells enables us to evaluate tissue damage after direct current epidural stimulation (Colmenárez-Raga et al., 2019). In the present study, no Iba1-reactive cells were identified, which demonstrates that our protocol of non-invasive stimulation is safe. Moreover, all changes described here resulted from electric stimulation and not from tissue damage.

### 4.1. Technical considerations

The ICA color space method is an experimental procedure developed for biometrical recognition with a more effective color representation (Tailor et al., 2000; Yin, 2002; Liu and Yang, 2009). Using this method, the density gradient of our images was converted into colors for a clearer separation between the background (yellow to brown) and the reaction product (magenta to white; see calibration bar in Figure 1). ICA conversion highlighted subtle differences in gray values, allowing us to distinguish c-Fos immunoreactivity in the cytoplasm (magenta in Figure 1).

MATLAB maps graphically represent normalized OD and the number of reactive neurons (see Section “Materials and methods”) assessed after a maximum entropy density threshold segmentation (ImageJ). Unlike ICA pseudocolor maps, which represent global tissue differences in gray levels, MATLAB maps show differences from immunoreactive segmented neurons, allowing to evaluate changes in distribution, size, and density of immunostaining at a cellular level. Therefore, both mapping approaches give complementary information to detect activation induced by electric stimulation protocols.

c-Fos is a neuronal activation marker which increases in response to a broad range of stimuli, including neuronal depolarization and firing, neurotransmitter release, synaptic stimulation, and changes in growth factor regulation. As such, c-Fos is a widely used tool for assessing electric stimulation effects on the brain (Ceccatelli et al., 1989; Bullitt, 1990; Krukoff et al., 1994). Although its induction can be regulated by many factors, increases in c-Fos immunoreactivity strictly reflect increases in cell protein synthesis (Sheng and Greenberg, 1990; Herrera and Robertson, 1996; Abe et al., 2001; Brivanlou and Darnell, 2002; Perrin-Terrin et al., 2016). Consequently, the final level of cytosolic c-Fos depends on the level of gene activation and RNA synthesis and on proteasome degradation (Jariel-Encontre et al., 1997; Salvat et al., 1999; Ferrara et al., 2003).

Cytosolic increases in c-Fos immunoreactivity have been detected in response to different transcription factors, such as TPA or G-SCF, in cell cultures (Higashi et al., 2004). Based on these findings, the increases in the cytosolic density of the reaction product (magenta in our ICA maps) observed in this study should be related to a heightened c-Fos synthesis. Therefore, the magenta shifts identified in cells, in our ICA maps, most likely reflect c-Fos overactivation.

### 4.2. Stimulation effects

Grossman et al. (2017) reported a higher c-Fos immunoreactivity in the dentate gyrus than in the cortex, suggesting that stimulation *via* temporally interfering electric fields primarily occurs in deeper rather than superficial areas of the brain. Notwithstanding interspecies differences (mice vs. rats), our results from a similar tTIS approach, corroborate the findings of these authors, showing a lower c-Fos activation in the cortex than in the dentate gyrus. Small changes in electrode montage and survival time for c Fos activation (40 vs. 90 min) with respect to the approach described by Grossman et al., may also explain differences in the topographical distribution of activation areas shown by us.

Our results also confirm the notion that tTIS activates deeper brain areas differently from superficial areas, especially considering that we examined anatomical changes in almost the whole brain and all its c-Fos positive cells.

Applying stimulation paradigms like those of Grossman et al. (2017), Rampersad et al. (2019) have recently reported the results of computational simulations in a murine head model, comparing AC and tTIS effects. In their mice model, in silico AC stimulation showed extensive voltage peaks homogenously affecting almost all superficial brain areas. In line with these results, our ICA pseudocolor maps of ES/AC sections showed extensive and homogeneous c-Fos activation in the cortex and hippocampus, but not in deep areas such as the brainstem or the diencephalon (Figure 3).

In their experimental model, however, tTIS evoked the strongest effect of the electric fields in any direction around the skull electrodes. Conversely, in both our ICA and MATLAB maps, the strongest effects were detected in the cortex, located just below the electrode coordinates, as shown by the decrease in neuronal c-Fos immunoreactivity with the increase in blood vessels’ walls. Thus, our results support the hypothesis that the decrease in neural activation induced by low-frequency (10 Hz) tTIS topographically matches the distribution intensity of electric fields, as predicted by Rampersad et al. (2019). Asymmetry in brain activation induced by tTIS shown by us (Figure 6D), is likely a consequence of the confluence of the two interfering currents inside the brain and is originated by the placement of both electrodes on one side of the skull. Despite the current flow inside the brain cannot be accurately predicted due to changes in tissue anisotropy, our results speak in favor of a repetitive directional interference amplification close to the right deep ventral parts of the brain. Therefore, future experiments comparing *in silico* and *in vivo* analysis, needs to be made after testing different electrode array configuration, to define more accurately predominant vectors of interference fields stimulation inside the brain.

Previous studies have analyzed the effects of neuronal activation or inhibition after alternating low-frequency current stimulation* in vivo*, but the biophysics of these processes and their net effects in connected neuronal networks remain elusive (Sandkühler et al., 1997; Durand et al., 2006; Cavarretta et al., 2014; Ye and Steiger, 2015; Ghasemi et al., 2019). After 1 week of sinusoidal epidural stimulation of the sensory parietal cortex in rats, with currents at different frequencies (20, 40, 60, and 100 Hz), c-Fos immunoreactivity was highest at 40 Hz and decreased at 60 Hz and 100 Hz, also suggesting a frequency-dependent effect on excitatory and inhibitory neurons (Ryu et al., 2019). These authors showed that stimulation at 20 Hz and 40 Hz are effective for inhibitory and excitatory cortical modulation, respectively. In rat brain slices, which included the subthalamic nucleus and its target, the globus pallidus, alternating current stimulations at 10 Hz and 100 Hz induced long-lasting depression and less frequently long-lasting excitation, respectively (Lavian et al., 2013). Although low-frequency currents tend to depress neuronal activity, in *in vivo* neuronal networks, in which the balance of excitation and inhibition may depend on the properties of the stimulus, the final effects of stimulation become unpredictable (Khatoun et al., 2017).

### 4.3. Future perspectives

In this study, c-Fos cytoplasmic immunoreactivity also increased in blood vessel cells and neurons after stimulation (ICA magenta; Figure 7). As explained above, cytosolic increases in this reaction product may reflect cell overactivation. Because the increase of c-Fos and GFAP immunoreactivity in large blood vessels is highly correlated with areas with decreased neuronal c-Fos immunoreactivity (i.e., close to the skull electrode coordinates) our results highlight the potential induction of hemodynamic changes (trophic effects).

Functional imaging of hemodynamics after intracortical stimulation *in vivo* using free optical-resolution photoacoustic microscopy (OR-PAM) demonstrated that the baseline diameters of blood vessels in the resting state can be altered by electrical stimulation, inducing vasodilatation or vasoconstriction depending on the current parameters (Tsytsarev et al., 2011). In a recent review of basic and clinical research, the authors emphasized that the main vascular effects occur after stimulation, depending on the anatomy and physiology of the blood vessels and on stimulation waveforms (Bahr-Hosseini and Bikson, 2021). In conclusion, the results from these studies, together with our findings, underscore the need to perform more specific experiments for analyzing hemodynamic changes and trophism after tTIS.

## 5. Conclusions

Stimulation *via* temporally interfering electric fields with sinusoidal currents of 2,000–2,010 Hz at 0.12 mA results in a global decrease in neuronal activation, as shown by c-Fos mapping across the brain. Such evoked neuronal activation depression affects most brain structures and is strongest at the electrode coordinates, decreasing gradually with the increase in the distance from the current source. Although tTIS affects the whole brain, asymmetric areas of activation suggest that tTIS has directional effects. The increased c-Fos immunoreactivity of blood vessels perivascular astroglia also provides evidence of potential vascular hemodynamic and trophic effects. Thus, our results indicate that tTIS may induce a combined effect on the trophic and metabolic activities of brain cells and significant changes in neuronal excitability.

## Data availability statement

The original contributions presented in the study are included in the article, further inquiries can be directed to the corresponding author.

## Ethics statement

The animal study was reviewed and approved by care and use of animals in biomedical research (Permit number: USAL- 685 - 2021) University of Salamanca, Spain.

## Author contributions

MM and JD-G designed the experiments. VC-B, IF, JD-G, AF, and MM performed the experiments and analyzed data. IL contributed by performing histological methods. VC-B and IF did the quantitative immunocytochemical study. All authors participated in the discussion of the experiments. MM wrote the article. All authors contributed to the article and approved the submitted version.

## Abbreviations

tTIS: transcranial alternating current and temporal interference
AC: alternating current
OD: optical density
ICA: independent component analysis
GFAP: glial fibrillary acidic protein
Iba1: ionized calcium-binding adapter molecule 1
DC: direct current
CSF: cerebrospinal fluid
fMRI: functional magnetic resonance imaging
SC: sham control
ES/tTIS: Electrical stimulation/ transcranial temporal interference stimulation
ES/AC: electrical stimulation/alternating current (non-interference) stimulation
SNR: Substantia nigra pars reticulata
LM: lateral mammillary nucleus
LG: lateral geniculate body
III V: third ventricle
ROIs: regions of interest
OR-PAM: optical-resolution photoacoustic microscopy
TeA: temporal associative
V2L: secondary visual cortex
EC: external cortex of the inferior colliculus
Sag: nucleus sagulum
IF: inferior colliculus
RSD: retrosplenial cortex
V1: visual primary cortex
CPu: caudate putamen.
